# Effect of Different Broad Waveband Lights on Membrane Lipids of a Cyanobacterium, *Synechococcus* sp., as Determined by UPLC-QToF-MS and Vibrational Spectroscopy

**DOI:** 10.3390/biology5020022

**Published:** 2016-05-23

**Authors:** Olimpio Montero, Marta Velasco, Aurelio Sanz-Arranz, Fernando Rull

**Affiliations:** 1 Centre for Biotechnology Development (CDB), Spanish Council for Scientific Research (CSIC), Francisco Vallés 8, Valladolid 47151, Spain; m.velasco@dicyl.csic.es; 2 Fisica de la Materia Condensada Department, University of Valladolid, Valladolid 47011, Spain; jausanz@gmail.com (A.S.-A.); rull@fmc.uva.es (F.R.)

**Keywords:** *Synechococcus* sp., UPLC-MS, vibrational spectroscopy, Raman, IR, lipids, pigments, light quality

## Abstract

Differential profile of membrane lipids and pigments of a *Synechococcus* sp. cyanobacterial strain cells exposed to blue, green, red and white light are determined by means of liquid chromatography and mass spectrometry or diode array detection. Raman and ATR-IR spectra of intact cells under the diverse light wavebands are also reported. Blue light cells exhibited an increased content of photosynthetic pigments as well as specific species of membrane glycerolipids as compared to cells exposed to other wavebands. The A_630_/A_680_ ratio indicated an increased content of phycobilisomes (PBS) in the blue light-exposed cells. Some differences in the protein conformation between the four light waveband-exposed cells were deduced from the variable absorbance at specific wavenumbers in the FT-Raman and ATR-FTIR spectra, in particular bands assigned to amide I and amide II. Bands from 1180 to 950 cm^−1^ in the ATR-FTIR spectrum suggest degraded outer membrane polysaccharide in the blue light-exposed cells.

## 1. Introduction

*Prochlorococcus* and the cyanobacterium *Synechococcus* are considered the most abundant photosynthetic organisms on Earth, and they both substantially contribute to chlorophyll biomass and primary production [1,2]. Ecological niches of marine *Synechococcus* members are coastal regions and mesotrophic open ocean surface waters [1], as well as water bodies with high to moderate suspended particulate matter. Scattering of light in a given water body is dependent upon different factors, including density and particulate matter amongst others, and it was experimentally measured to be inversely proportional to the wavelength power of 4.3 [3]. This fact leads to a strong backscattering on the water surface for incident blue wavelengths, whereas pure seawater has a high absorptivity at the red end of the spectrum and, thus, irradiance attenuation by pure water itself shifts the light spectrum within water towards the blue wavelengths as compared to the incident solar spectrum [4]. Likely bound to this feature, Ferris and Palenik [5] reported that *Synechococcus* isolates at different depths have specific light requirements for optimal growth. However, vertical movement of cells within the water column exposes them to varying irradiance intensities from diverse wavelengths and cells must therefore be able to acclimate their photosynthetic performance to such changes for growth and survival [6].

As for any photoautotroph, *Synechococcus* cell metabolism is primarily dependent on photosynthesis, whose efficiency becomes strongly bound to a balanced coupling between light absorption through the light harvesting complexes (LHCs) and primary reactions at the reaction centers (RCs). In this regard, it has been pointed out that an imbalance between photosystem composition and the available light quality and/or quantity can result in the generation of reactive oxygen species (ROS), which can lead to nucleic acid and protein damage as well as lipid peroxidation and, consequently, to cell death [6,7]. Therefore, in order to limit such an imbalance, almost all cyanobacterial cells drive changes in the composition and conformation of their photosynthetic apparatus to cope with varying environmental conditions, in particular light intensity and quality. The capacity of cyanobacterial cells to modify their pigmentation contents and the phycobilisome/chlorophyll ratios in particular, is termed complementary chromatic adaptation (CCA) [8]. Phycobilisomes (PBSs) are the major light-harvesting complex in cyanobacteria and their composition for a particular cyanobacterial species is bound to the varying light wavelengths the cell has to face over a day cycle. Hence, most research regarding CCA has been and is primarily focused on how the synthesis of the three main pigments of the PBS—phycoerythrin (PE), phycocyanin (PC) and allophycocyanin (APC)—is regulated [9]. Through diverse studies it has been shown that the photocontrol of PE and PC levels is mainly led by regulation of the cpeBA and cpcB2A2 operons’ transcription [9] (and references therein). By measuring the action spectra, Vogelmann and Scheibe [10] established that green light (G, 550 nm) maximizes PE synthesis, whereas red light (R, 640 nm) maximizes PC synthesis. Growth rates of *Synechococcus* sp. PCC7002 cultivated under monochromatic 680 nm light have been shown to be about 30%–40% lower than the growth rates under monochromatic 630 nm light or a combination of both wavelengths irrespective of the 630:680 ratio [11]. However, the quantum yield of photosystem II (Y_II_) and the relative maximal electron transfer rate (rETRmax) were 30 and 25% lower, respectively, for the monochromatically irradiated cultures at 630 nm than at 680 nm.

Chlorophyll *a* (chla) absorbs light photons at both extremes of the visible spectrum. Even though blue light may trigger photoinhibition because of its highly energetic photons, blue light seems to be necessary for the coordination of photosynthesis with other cellular processes through specific photoreceptors [6,9]. Indeed, two families of cyanobacteriochromes (CBRCs) that detect blue light to near UV (330–450 nm) are implicated in CCA regulation [12,13]. Furthermore, in aquatic systems, blue light reaches deeper layers and is therefore the main wavelength range available for photosynthesis. The *mpeZ* gene that encodes a phycoerythrobilin lyase-isomerase is more expressed under blue light than under green light [14].

Extensive research has been and is devoted to the underlying mechanisms involved in CCA regarding changes in PBS composition and organization as well as how these changes are coordinated with concurrent variations in photosystem composition and content. However, little attention has been paid to the composition and conformation of the thylakoid membrane under different quantity and quality light situations. Thylakoid membrane has been shown to be a dynamic network [15] that is likely to vary its composition and conformational state as necessary to accommodate the particular pigment/protein status triggered by CCA. Indeed, Montero *et al.* [16] showed that subtle changes in glycerolipid composition are observed when *Synechococcus* sp. cultures are transferred from low to high light.

A constraint for architectural and conformational studies of biomembranes is imposed by the non-crystalline structure of the lipid structure where proteins are embedded. Applications of Raman and Fourier-Transform Infrared (FT-IR) spectroscopies are being developed to characterize biomolecules as well as their interactions even in intact cells [17,18]. The Raman spectra of diverse biomolecules as well as of bacteria and fungal spores were reported by De Gelder *et al.* [19]. Nonetheless, the interpretation of both Raman and FT-IR spectra of intact cells is hard since multiple biomolecules contribute to signals and the vibrational state of a given chemical bond may be altered by the microenvironment.

In this study, pigments and membrane lipid contents along with FT-Raman and ATR-FTIR spectra of intact *Synechococcus* cells exposed to blue, green and red light are compared with those measured in white light-exposed cells. The *Synechococcus* strain used in this study was previously shown to be closely related (99% homology of the 16S rDNA) to the model strain *Synechococcus* sp. PCC7002 [20].

## 2. Materials and Methods

### 2.1. Experimental Setup

Stock cultures of the cyanobacterium *Synechococcus* sp. strain 01/0202 (Microalgae Culture Collection of the Institute for Marine Sciences of Andalusia (ICMAN, CSIC, Spain) were grown at 20 °C and about 45 µmol quanta m^−2^ s^−1^ provided by daylight fluorescent tubes in a culture chamber as in Montero *et al*. [16]. About 5 mL of the stock cultures were transferred to 25 mL of fresh medium in Petri dishes covered with transparent (white light), blue, green or red plastic filters. They will be further designated as W, B, G and R cultures, respectively. Cell suspensions were then left under the same illumination and temperature as the stock cultures for up to 48 h. Culture samples (2 mL) were withdrawn at 24 h and 48 h for methanolic extraction. After centrifugation (3212 g, 4 °C, 10 min) of the cell suspension, the supernatant was discarded and the pellet was washed with 0.9% NaCl. Next, the pellet was recovered after a new centrifugation and 1 mL 100% methanol was added. The mixture was then sonicated for 1 min and the supernatant collected after centrifugation. The methanolic extract was then kept at −20 °C under nitrogen atmosphere for less than 24 h until analysis. An additional sample volume of 0.5 mL was also withdrawn for immediate UV-Vis spectrum measurement at each sampling time. Independent experiments under the same conditions were carried out for sampling in the vibrational spectroscopy (Raman and Infrared) measurement experiments. In these experiments, cells were collected by filtration on a nylon membrane over a time lower than 2–3 min. The experimental setup was repeated by triplicate or duplicate in the vibrational spectroscopy experiments.

### 2.2. High-Performance Liquic Chromatography with Photodiode Array Detection (HPLC-DAD) Measurements

Photosynthetic pigments in the methanolic extract were analyzed by high-performance liquid chromatography with photodiode array detection (HPLC-DAD) using the same chromatographic method as in Montero *et al*. [16,20], but UV-Vis spectra were recorded for each peak over the range 350–750 nm in the present study. A FINNIGAN SURVEYOR PLUS chromatography system (Thermo Scientific) equipped with Quaternary LC Pump, Autosampler and PDA detector was used for HPLC-DAD measurements. Pigments were identified according to retention time and UV-Vis spectrum. Relative quantification was done from the peak area in the maximal absorption wavelength (MaxPlot) chromatogram.

### 2.3. Ultra-Performance Liquid Chromatography (UPLC)-Quadrupole Time-of-Flight Mass Spectrometry (QToF-MS) Measurements

The methanolic extract was also analyzed by ultra-performance liquid chromatography coupled to quadrupole time-of-flight mass spectrometry (UPLC-QToF-MS). An Acquity™ UPLC System (WATERS, Manchester, UK) equipped with an automatic injector (Sample Manager) and a binary solvent pump (Binary Solvent Manager) was used for liquid chromatography. The output of the liquid chromatographer was connected to a SYNAPT G2 HDMS mass spectrometer (Waters, Manchester, UK), with time-of-flight analyzer (QToF) and electrospray ionization source (ESI). Compounds were eluted at a flow rate of 0.35 mL/min using the gradient that follows: initial, 100% A; 1 min, 100% A; 2.5 min, 20% A; 4 min, 20% A; 5.5 min, 0.1% A; 8.0 min, 0.1% A; 10 min, 100% A, and this was kept isocratic for 2 min to recover initial pressure before next injection. Solvents were (A) methanol:water:formic acid (Met:H2O:FA 50:50:0.5, v/v/v) and (B) Met:ACN:FA (59:40:0.5, v/v/v), both with 5 mM ammonium formate. The chromatographic column was an Acquity UPLC BEH HSS T3 100 × 2.1 mm, 1.7 µm p.s., with a 10 × 2.1 mm precolumn (vanguard column) of the same stationary phase. Samples were analyzed with positive and negative ionization using a MS^E^ method [21]. Compounds were identified by the *m/z* value, the elemental composition compatible with the isotopic distribution and relative retention time as well as specific fragments from MS^E^. Quantification was carried out by integrating the area of the chromatographic peak obtained in the extracted ion chromatogram (EIC) for each compound with the QuantLynx application available with the instrument software (Waters, Manchester, UK).

### 2.4. Fourier-Transform Raman (FT-Raman) and ATR-Fourier-Transform Infrared (ATR-FTIR) Spectroscopies

For FT-Raman and ATR-FTIR spectroscopic analysis of *Synechococcus* cells, a 5 mL volume of cell suspension was filtered through PTF membranes and cell biomass immediately placed on special devices used for the spectroscopic measurements. For ATR-FT-IR measurements, a Perkin Elmer Spectrum 100 FT-IR spectrometer with Universal ATR Sampling Accessory was used. Spectral resolution was 4 cm^−1^ and 16 cumulative spectra were acquired for each measurement. FT-Raman spectra were acquired with a FT-Raman Bruker RFS100/S equipped with a Láser Klastech, Senza series (1064 nm, 500 mW) and a CCD Bruker D418-T (range 851–1695 nm). The diameter of the LASER spot over the sample was 1000 µm. Other parameters were 10 mm slit, scan rate of 1.6 KHz, and the number of accumulations was 1024.

### 2.5. Statistical Analyses

Statgraphics Plus version 5.0 was used for two-way ANOVA comparisons of relative content data (main variable) for every compound class (MGDG, DGDG, SQDG, and PG) with compound and filter as factors within each exposure time (24 h or 48 h) or compound and time as factors. Paired Student’s *t*-test was done with EXCEL software (Microsoft Office 2010).

## 3. Results and discussion

### 3.1. UV-Vis Spectra

*In vivo* absorption spectra of *Synechococcus* sp. cultures after 24 h and 48 h of growth under white light (transparent filter, W), blue light (blue filter, B), green light (green filter, G) and red light (red filter, R) are shown in supplementary Figure S1. The ratio of the absorbance at 632 nm to the absorbance at 680 nm (A_630_/A_680_), which may be considered as indicative of the relative proportion between phycocyanin (PC) and chlorophyll *a* (chla), was higher in the B cultures than in the other cultures, with the lowest value being shown by the W culture (Table 1). Significant differences between the values after 48 h and 24 h of filtered light exposure were found for all the cultures except for the R culture. This latter culture exhibited significant differences with the other cultures for the 24 h exposure period, but only with the B culture in the 48 h exposure period. Values of the A_630_/A_680_ ratio were significantly different between the W, B and G cultures after both 24 h and 48 h of growth. Using light-emitting diodes (LEDs), Kim *et al.* [22] reported a rise in PBSs of a *Synechococcus* sp. strain under green and blue light as compared to white and red light. In *Nostoc* *sphaeroides,* Kützing PBS content also was higher under blue light than under red light supplemented with white light, though the PC content was augmented under red light [23]. These results are in opposition to PBP bleaching after only 45 min of exposure to blue light, as bleaching requires 120 min and up to 24 h under green and red light exposure, respectively [24]. However, this discrepancy can be rationalized in terms of a probable imbalance between excessive photon fluence rate (PFR) of the blue wavelengths and the turnover rate of the photosynthetic apparatus to accommodate its molecular composition to such PFR in the aforementioned study [24].

The absorption spectra recorded for the methanolic extracts of the cultures grown under exposure to the different light filters are shown in Supplementary Figure S2. The ratio of the absorbance at 475 nm to the absorbance at 665 nm, which may be considered as indicative of the carotenoid/chlorophyll *a* (chla) ratio, is depicted in Table 2 for the different cultures. This ratio was higher for the W culture at both 24 h and 48 h. Significant differences were found between the W (transparent filter), G and R cultures at 24 h, whereas at 48 h, significant differences were found between the W and G cultures, the W and R cultures, and the B and R cultures. These results are consistent with the fact that carotenoids absorb within the blue wavelength range, whereas red wavelengths are mainly absorbed by chla. Carotenoids are also scavengers of radicals, which are likely increased under blue light due to over-oxidized chla.

### 3.2. UPLC-Mass Spectrometry (MS) Analysis

**Figure 1:**
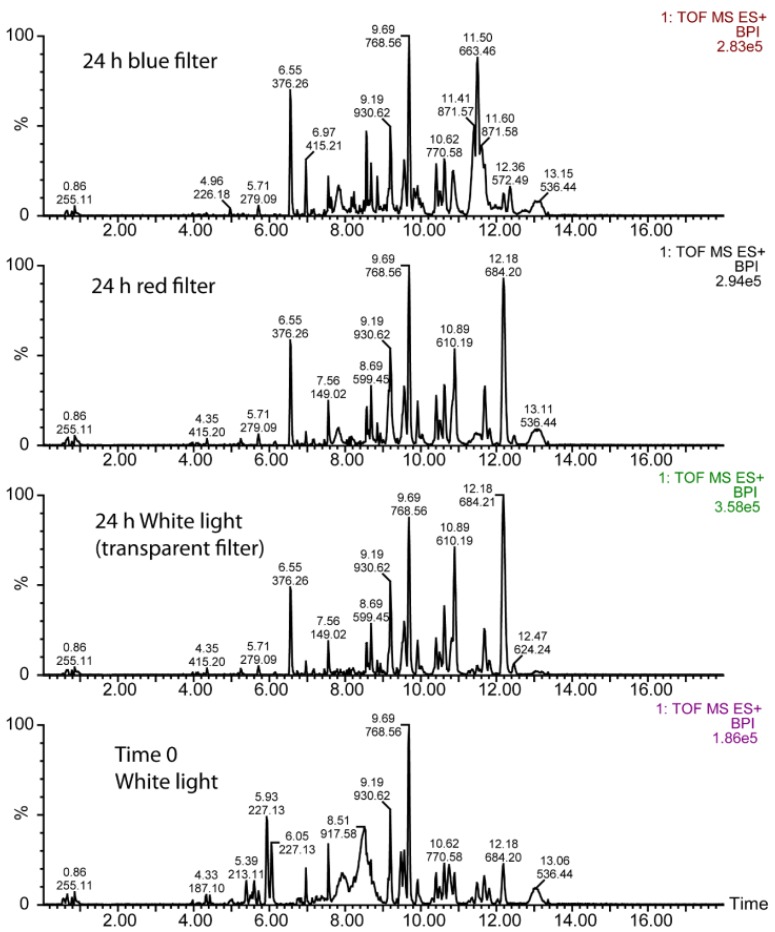
Typical base peak intensity (BPI) chromatograms obtained for the methanolic extract after UPLC-MS analysis of the culture covered with a blue filter for 24 h (upper panel), the culture covered with a red filter for 24 h (second panel down from top), the culture covered with a transparent filter for 24 h (second panel up from bottom), and the stock culture grown under white light and used for the inoculum of the transparent, red and blue filter cultures (lower panel). The numbers above each chromatographic peak indicates the retention time (up) and the *m/z* predominating in that peak (down). Right legends: 1. TOF MS ES+, MS with electrospray positive ionization; BPI, base peak intensity; number, intensity to which 100% is fitted.

Typical base peak intensity (BPI) chromatograms obtained for positive ionization in the UPLC-MS analysis of the methanolic extract of cells from the stock culture (time 0) and the W culture (transparent filter), B (blue filter) and R (red filter) cultures after 24 h of exposure to the filtered white light are illustrated in Figure 1. A prominent peak raised by *m/z* 684.20 was observed in the W and R cultures, although it was absent in the B culture. Conversely, a prominent peak raised by *m/z* 663.45 in the B culture was clearly diminished in the W and R cultures.

Current components of the membrane chemical profile of *Synechococcus* species [16,20,25] were detected as the [M+NH4]^+^ and [M+Na]^+^ adduct ions in positive mode, and the [M+HCOOH-H]^−^ plus [M+HCl-H]^−^ adduct ions for monogalactosyl^−^ and digalactosyldiacylglycerol (MGDG and DGDG, respectively), and the [M-H]^−^ ion for sulfoquinovosyldiacylglycerol (SQDG) and diacylphosphatidylglycerol (PG) in negative mode. Chlorophyll *a* (C55H72N4O5Mg) was detected in negative mode as the [M+HCOOH-H]^−^ adduct ion with *m/z* 937.5328 (∆ 1.1 ppm), the fragmentation spectrum rendering the ions with *m/z* 891.5251 [M-H]^−^), *m/z* 539.1964 ([M-C23H43O2-H]^−^, C23H43O2 = phytylpropionate), and *m/z* 525.1774 ([M-C23H43O2-CH2-H]^−^). In positive mode, chlorophyll *a* (chla) was detected as the [M]^+^ radical ion with *m/z* 892.5352 (∆ −0.1 ppm), the predominant [M-Mg+3H]^+^ ion with *m/z* 871.5709 (∆ −3.2 ppm), and lesser abundant radical ions [M+2NH4]^+^ and [M+NH4+Na]^+^ with *m/z* 928.6005 and 933.5565, respectively. The *m/z* 871.5709 was also detected as the [M+H]^+^ ion of the pheophytin *a* but without the accompanying ions and to a different retention time. Species of the glycerolipids that currently constitute the *Synechococcus* membrane chemical profile were identified as previously reported along with new species (Table 3). Identification of the acyl substituents was conducted by the correspondent [M-H]^−^ ion in the fragmentation spectrum under negative ionization. Furthermore, for MGDG and DGDG species, fragments (MS^E^) obtained under positive ionization of the remaining lysoMGDG and lysoDGDG after losing one of the acyl chains esterifying the glycerol backbone in addition to the polar head were identified as acyldesoxyglycerol (C_n_H_m_O_3_, where n is the carbon number of the acyl chain plus 3C and m is the number of hydrogens of the acyl chain plus 6). As well, the [M+Na]^+^ could be clearly observed in the MS^E^ spectrum.

**Figure 2:**
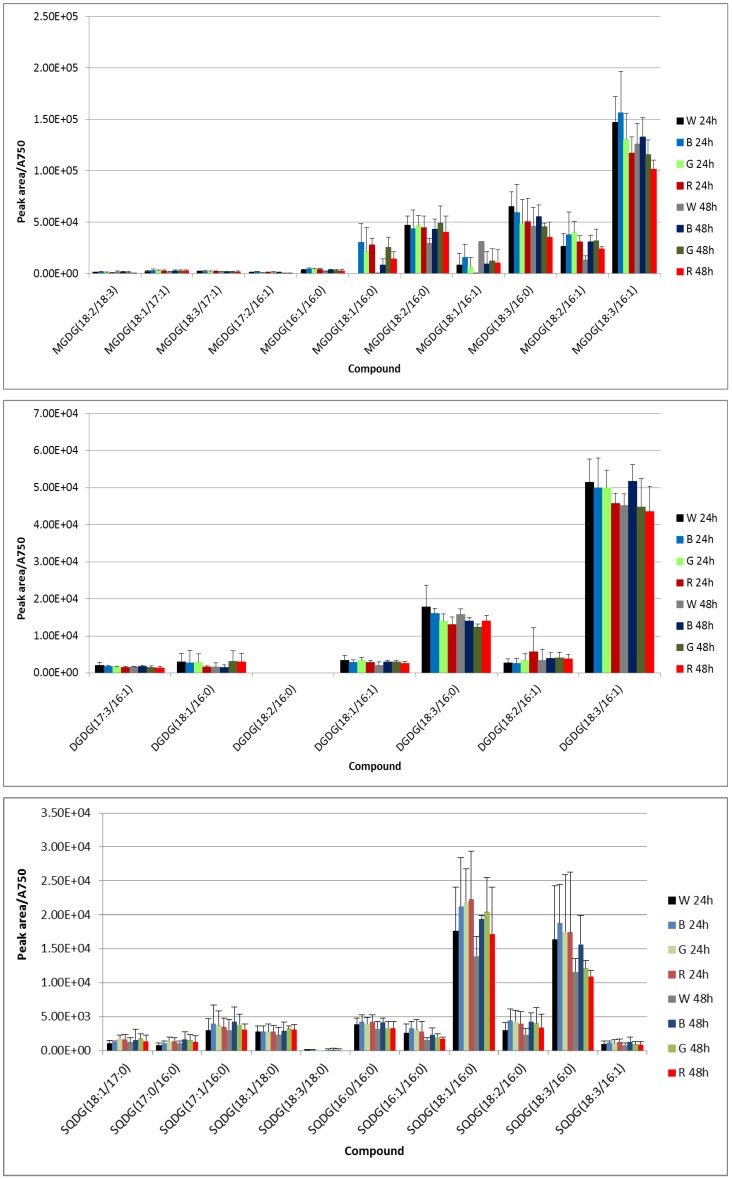

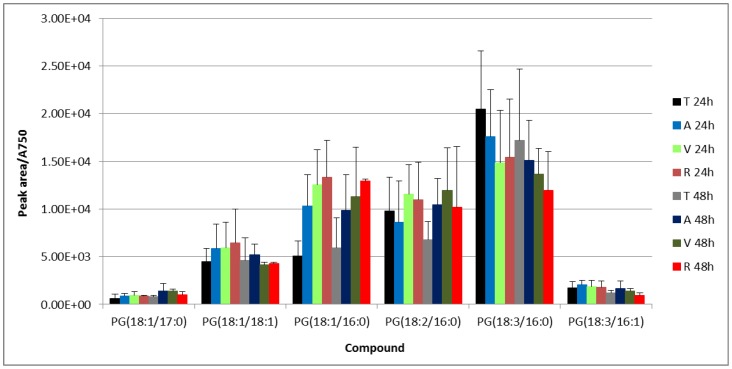
Relative content normalized to the absorbance at 750 nm of the monogalactosyldiaylglycerol (MGDG) species, digalactosyldiacylglycerol (DGDG) species, sulfoquinovosyldiacylglycerol (SQDG) species, and phosphatidyldiacylglycerol (PG) species as measured by UPLC-MS with positive (MGDG and DGDG) or negative (SQDG and PG) electrospray ionization from a methanolic extract of *Synechococcus* sp. cells. Glycerolipid nomenclature used in this study is that recommended by the LipidMaps consortium (http://www.lipidmaps.org/), where the acyl chains esterifying the *sn*-1 and the *sn*-2 positions of the glycerol backbone are located in the first and second places within the brackets (*sn*-1/*sn*-2), and the polar head group (phosphatidylglycerol, sulfoquinovosyl, galactosyl and digalactosy) is assumed to esterify the *sn*-3 position of the glycerol backbone.

According to the relative quantification, predominant membrane glycerolipids were MGDG (18:3/16:1), MGDG (18:3/16:0), MGDG (18:2/16:1), DGDG (18:3/16:1), SQDG (18:3/16:0), SQDG (18:1/16:0), PG (18:3/16:0), PG (18:2/16:0), PG (18:1/16:0) and PG (18:1/18:1) (Figure 2). Two-way ANOVA showed statistical differences (*p* < 0.001) between MGDG (18:3/16:0), MGDG (18:2/16:1) and MGDG (18:3/16:1), and between them and the remaining MGDGs except between MGDG (18:2/16:1) and MGDG (18:2/18:3). Only DGDG (18:3/16:1) was found statistically different from the other DGDGs at 24 h but all of them statistically different to each other except DGDG (17:3/16:1) and DGDG (18:2/16:1) on the one hand, and DGDG (18:1/16:0) and DGDG (18:3/16:0) on the other hand at 48 h. No statistical differences at both 24 h and 48 h were found between SQDG (17:1/16:0), SQDG (16:0/16:0) and SQDG (18:2/16:0), between SQDG (18:1/16:0) and SQDG (18:3/16:0), and between the other SQDGs except SQDG (18:3/18:0) and SQDG (18:3/16:1). All PG species were statistically different between them with the exception of PG (18:1/17:0) and PG (18:3/16:1) as well as PG (18:1/16:0) and PG (18:2/16:0).

Using data from negative ionization, significant statistical differences (*p* < 0.05) after paired *t*-test were shown at 24 h for MGDG (18:1/16:0) between B-W and R-W cultures, and at 48 h for MGDG (18:1/16:0) between G-W cultures, for DGDG (18:3/16:1) between B-W cultures, and for SQDG (18:1/16:0) between B-W cultures. When data from positive ionization were compared, significant statistical differences (*p* < 0.05) after paired *t*-test were found for MGDG (18:1/16:0) between B-W, R-W and G-W cultures, for MGDG (18:3/16:1) between R-W cultures, for DGDG (18:3/16:0) between B-G and B-R cultures; for SQDG (18:3/16:0) between B-G cultures, and for PG (18:3/16:0) between G-W cultures; and at 48 h for MGDG (18:2/16:1) between B-W and R-W cultures, for MGDG (18:3/16:1) between B-R cultures, and for DGDG (18:3/16:0) between B-G and G-W cultures. The content of DGDG (18:2/16:1) and DGDG (18:3/16:0) decreased after 48 h of exposure as compared to their content after 24 h of exposure (*p* = 0.045 and 0.028, two-way ANOVA), with the blue light-exposed culture exhibiting the highest content after 24 and 48 h of growth (Figure 2). A similar trend was observed for DGDG (18:3/16:1) although no significant differences were shown by this compound. All the cultures had a reduction (*p* = 0.038, two-way ANOVA) in the SQDG (18:3/16:0) content after 48 h of growth as compared to the content after 24 h of growth, with an apparent trend of the content in the B culture being higher than in the other cultures (although only significantly different between the B and G cultures as indicated above). PG (18:1/16:0) showed a content in the W culture significantly lower than in the other cultures after 24 h (*p* = 0.014, 0.010 and 0.008 for the B, G and R cultures, respectively), but only in the R culture after 48 h of light exposure (*p* = 0.030). Conversely, PG (18:3/16:0) was predominant in the W culture in comparison to the other cultures under both exposure periods (only significant differences were shown between the G and W cultures, as indicated above), and the R culture exhibited a notable reduction in the content of the glycerolipid after 48 h of exposure as compared to the 24 h of exposure period (but with no significant statistical differences) (Figure 2, lower panel).

Changes in the membrane glycerolipid composition are likely to point out diverse conformational states regarding photosystem proteins and PBS accommodation. Variations in the content of any glycerolipid with a particular acyl chain configuration but not all may be related to the specific functions that have been attributed to every membrane glycerolipid, which might be in accordance with the changes in the composition of the photosynthetic apparatus triggered by the CCA. Membrane glycerolipid functions, in addition to a structural one, can be assumed to arise from their polar head group facing the hydrophilic side and the acyl chain unsaturation. The polar head group is likely involved in particular functions like pH regulation in both stroma and lumen, which leads to the anionic PG and SQDG being mainly located at the stromal side, whereas neutral DGDG species predominate at the lumenal side; MGDG is distributed at both sides of the thylakoid membrane [26,27]. Acyl chain unsaturation contributes to membrane thickness through *cis/trans* isomerism and to membrane fluidity by increasing the number of double bonds [28]. It has been shown that cyanobacterial state transitions are dependent on membrane fluidity because redistribution of PBS-absorbed energy requires mobile photosystems II and I [29,30]. In addition, it was found that the higher the unsaturation of membrane lipids, the higher the turnover rate of photosystem II D1 protein [28].

**Figure 3:**
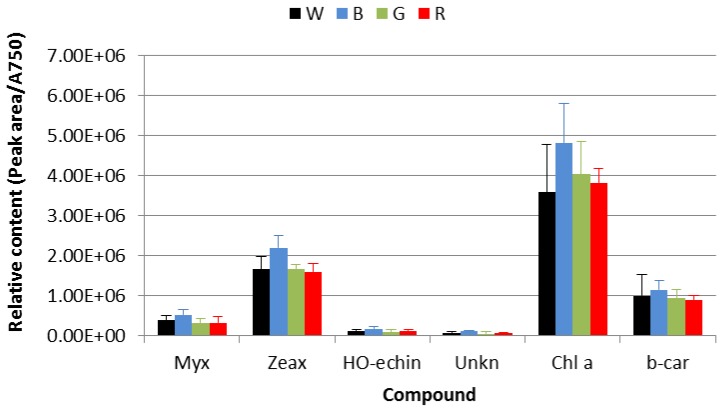
Relative content normalized to the absorbance at 750 nm of the main photosynthetic pigments as measured by HPLC-DAD from a methanolic extract of *Synechococcus* sp. cells. Legend: W, transparent filter; B, blue filter; G, green filter; and R, red filter. X-axes legend: Myx, myxoxanthophyll; Zeax, zeaxanthin; HO-echin, hydroxy-echinenone; Unk, unknown (echinenone related); Chl a, chlorophyll *a*; b-car, β-carotene.

Even though significant statistical differences could only be found in a reduced number of species, a general trend of increased content of the more unsaturated species in the B culture may be depicted from the results of this study. It should be taken into consideration that the irradiance used in this study is rather low, and pronounced effects are not to be expected. The rise in unsaturated species might be related to the increased PSII/PSI ratio that has been reported under monochromatic blue light [11], as the increased content of chla suggests (see below, Figure 3). Blue light is known to elicit non-photochemical quenching (NPQ) in cyanobacteria, with concurrent heat dissipation and likely D1 damage, a mechanism that proceeds through absorption by the orange carotenoid protein (OCP) which once activated binds to the PBS core [31,32,33]. In 48 h grown cells, the (18:3/16:1) acyl chain profile, which is the major one in MGDG and DGDG, apparently rose under blue light as compared to the other wavebands, a fact that might be directly bound to the need for an enhanced membrane fluidity that facilitates D1 turnover. Furthermore, membrane spanning would be necessary to accommodate the higher number of PBSs that seems to arise under blue light, which may be accounted by a cone-like glycerolipid such as MGDG [34], although the content of DGDG has to increase as well in order to keep membrane stability [35]. Conversely, MGDG (18:3/16:1) was significantly reduced under red light, which is mainly absorbed by PC-enriched PBPs, likely suggesting that light harvesting and charge separation were properly balanced with minimal detrimental effects on D1 protein and, hence, no enhanced membrane fluidity was necessary. Dependence on MGDG species content has been reported for violaxanthin de-epoxidase activity in eukaryotic cells [36]. DGDG (18:3/16:0) was significantly reduced in the G and R cultures after 24 h, but only in the G culture after 48 h of light exposure. Green light is mainly absorbed by PE; DGDG (18:3/16:0) might therefore play a particular role in regulating PBS association with the thylakoid membrane depending on their PE/PC composition.

PG, and to a certain extent SQDG as well, species were shown to play essential roles in cyanobacterial photosynthesis [37,38,39,40]. Liu *et al.* [41] reported that in three cyanobacterial strains, including *Synechococcus* sp. PCC7002, PG (18:3/16:0) content dropped with culture age, which was accompanied by a concurrent rise in the PG (18:1/16:0) species, and a general decrease in unsaturation during the stationary phase. PBSs are associated with the thylakoid membrane at the stromal side where these anionic glycerolipids are mainly located [26]. Therefore, variations in species of PG and SQDG are likely to be related to PBS association with the thylakoid membrane through the linker polypeptide. However, PG and SQDG species with the acyl chains (18:1/16:0) and (18:2/16:0) were apparently augmented in the B, G and R cultures as compared to the W culture, thus suggesting no waveband dependence. Conversely, the most abundant species PG (18:3/16:0) and SQDG (18:3/16:0) were apparently diminished in the B, G and R cultures as compared to the W culture (to a lesser extent in the B culture for the SQDG species). This feature might have to do with a heterogeneous distribution throughout the thylakoid membrane with specific membrane domains (lipid rafts) being particularly enriched in any given glycerolipid species. Indeed, it has been proposed that membrane protein function may be influenced by specific lipids [42,43,44]. According to photosystem location within the thylakoid membrane, some photosystem I proteins are rather close to the leaflet facing the stromal side, where PG and SQDG species are more abundant and, thus, the apparent increase in the (18:1/16:0) and (18:2/16:0) species of PG and SQDG may be bound to variations in PSII/PSI ratio and/or PBS movement regarding the light-state transition mechanism. Additionally, it should be taken into consideration that the subtle changes observed in this study may also stem from the cytoplasmic membrane compensating for those related to the thylakoid membrane. Hence, more specific and thorough experiments regarding lipase and desaturase activity as well as membrane fractionation and a higher irradiance exposure are necessary to improve these results.

### 3.3. HPLC-DAD Pigments

Main photosynthetic pigments were quantified as the peak area determined from the HPLC-DAD measurements of the methanolic extract and normalized to the absorbance at 750 nm for each sample (Figure 3). The culture grown under the blue filter (B) exhibited a pigment content significantly (Student’s paired *t*-test) higher than the other cultures with the exception of chlorophyll *a* (Chla) with respect to the red filter-grown culture (R) and β-carotene with respect to the transparent filter-grown culture (W). Significant differences between the other cultures were only found for myxoxanthophyll in the comparison of the R and G cultures against the W culture (Student’s paired *t*-test). The higher content of zeaxanthin and myxoxanthophyll in the B cells may be motivated by demand of increased heat dissipation and antioxidant activity [45,46].

### 3.4. Raman and Attenuated Reflectance Infrared (ATR-IR) Spectroscopy

**Figure 4:**
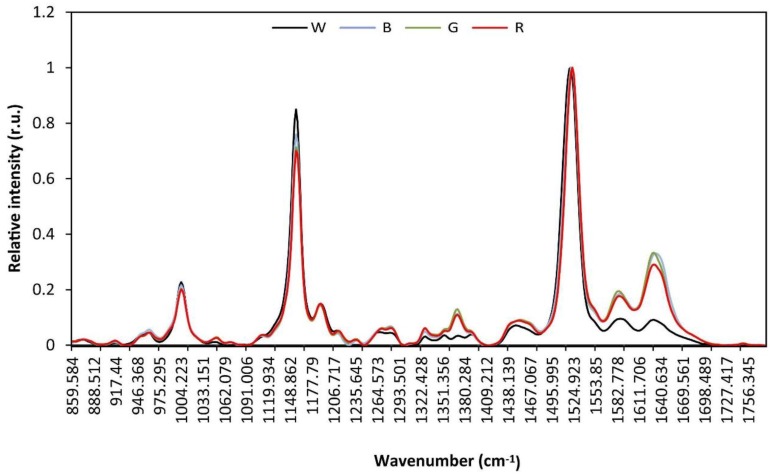
Raman spectra of intact *Synechococcus* sp. cells exposed to blue (B), green (G), red (R) and white (W) light.

**Figure 5:**
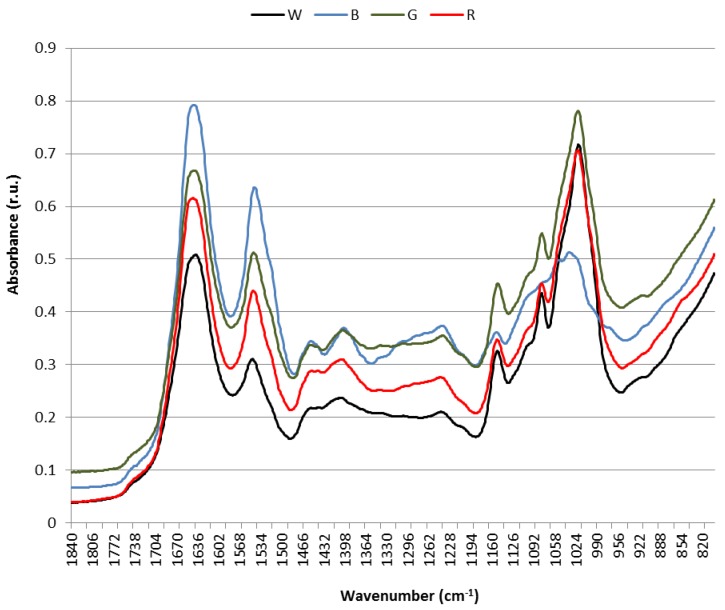
Attenuated reflectance Fourier-Transformed Infrared spectra (ATR-FTIR) of intact *Synechococcus* sp. cells exposed to blue (B), green (G), red (R) and white (W) light.

Varying absorbance at certain frequencies between the B, G and R cultures and the W culture were shown in the FT-Raman and ATR-FTIR spectra (Figure 4 and Figure 5). Most studies on Raman and IR spectroscopies have been carried out in membrane model systems. It is therefore not easy to interpret spectra of intact cell membranes [17].

Main differences in the Raman spectra arose in the region from 860 to 1710 cm^−1^ (Raman spectrum of each replicated sample is shown in Supplementary Figure S4). Less absorbance at specific frequencies were depicted by the culture under the transparent filter (W culture), whereas cultures under the blue, green and red wavebands displayed a more similar spectrum (Figure 4). Even though a more thorough analysis of Raman spectrum is devoted by deconvolution for accurate band assignment, a little shift towards higher wavenumbers of the peak at 1631 cm^−1^ in the B culture with regard to the other cultures may be appreciated. The band at 1630–1640 cm^−1^ has been ascribed to amida I, β-sheet of peptides [17], which may suggest that conformation of some proteins are changed in the B, G and R cultures with regard to the W culture, and particularly in the B culture. Otherwise, a broad band at 1630 cm^−1^ has been ascribed to ν(C=O) stretching of amide and carboxylic groups [19], and the slight shift towards higher wavenumbers in the B culture could then be indicative of an increase in the number of double bonds and/or their proximity to the C=O group.

A band around 1585 cm^−1^ was less intense in the W culture than in the other cultures with a slight shift towards lower wavenumbers in the latter ones. Even though this band cannot be ascribed to a particular vibrational frequency, it might be associated with stretching vibrations of CO_2_^−^, ν(CO_2_^−^) [47], possibly being interpreted as loss or degeneration of any undefined chemical group in G cells. The band around 1450 cm^−1^ may also correspond to that vibration [19] but it has also been ascribed to an increased vibration motivated by -CH2- bending, a fact that could be associated with a different membrane conformation and/or phase in the B, G and R cells as compared to W cells. It is noteworthy that the maximum of this band is slightly shifted towards a lower wavenumber in the W culture, which may suggest a different degree of hydration of the glycerolipid acyl chain C=O group in this culture in regard to the other cultures [17]. The region from 1315 to 1400 cm^−1^ particularly exhibited differences between cultures. Whereas the spectrum in this region is well resolved, yielding four peaks in the W cells, the rise in intensity of the peak centered around 1370 cm^−1^ in the other cells turns the peaks centered at 1349 and 1391 cm^−1^ into shoulders of the 1370 cm^−1^ peak. Peaks within this region are currently ascribed to C-H bond deformation, δ(CH), which could be derived from differential acyl chain tilt. The weak band at 1050 cm^−1^, ν(C-C) stretching, would underpin such assumption. Indeed, it was demonstrated that in bacterial membranes, peptides that are not adsorbed alter dehydration of C=O groups and formation of peptide-membrane hydrogen bonds [48], which may lead to these peaks arising from interactions between PBS and associated thylakoid membrane leaflet that induce changes in acyl chain topology.

The band peaking at 1282 and 1272 cm^−1^ can be ascribed to amide III due to the -N-H bend and C-N stretch [17]. Nonetheless, an intense band at 1273 cm^−1^ has been also assigned to deformation of CH unit [47], and weak bands at 1280 and 1270 cm^−1^ were shown in the β-carotene Raman spectrum [19]. Because there is a slight trend towards increased peaking at 1282 cm^−1^ in the G and B cells from the peaking at 1272 cm^−1^ in the W cells, with an intermediate situation in R cells, this band may correspond to the tetrapyrrole ring of phycobilins as the A_630_/A_680_ ratio suggests a similar situation. The band centered at 1230 cm^−1^ and the weak band at 1112 cm^−1^, ν(C-N), would be in agreement with such an assignment.

The IR spectra showed a slightly different picture to that of Raman spectra though there was coincidence in vibrational frequency assignment. Main differences between cultures were depicted in the wavenumber region from 1800 to 850 cm^−1^ (Figure 5). Contrary to Raman spectra, IR spectra showed variations between samples within the same culture (see Supplementary Figure S5); therefore, paired *t*-test was carried out between the different cultures to find out whether differences at relevant bands had statistical significance (see Supplementary Tables S1 and S2). The W culture did not show significant differences for the band at 1152 cm^−1^ with the B and G cultures, for the band at 1080 cm^−1^ with the B, G and R cultures, nor for the band at 1020 cm^−1^ with the R culture; the B and G cultures showed significant differences for the bands at 1152, 1103, 1080 and 1020 cm^−1^; and the R culture showed significant differences with the B and G cultures for the bands at 1453 and 1240 cm^−1^. In general, absorbance intensity at wavenumbers between 1700 and 1340 cm^−1^ exhibited a decreasing trend B > G > R > W, with defined bands peaking at about 1643, 1545, 1453 and 1401 cm^−1^ although the peak at 1453 cm^−1^ progressively disappeared from B to W. Conversely, in the wavenumber range from 1190 to 950 cm^−1^, the B cells showed a differential spectrum shape with substantially reduced absorbance as compared to the other cells, and with the G cells exhibiting maximal absorbance, they followed by R and W cells with similar absorbance intensity. Bands peaking at 1152, 1080 (with a shoulder at 1100) and 1020 cm^-1^ could be observed. The band peaking at 1643 cm^−1^ can be assigned to amide I, α-helix [17] although it has also been associated with ν(C=C) stretch, band II. The vibrational frequency at 1545 cm^−1^ is also assigned to either ν(-CO) stretch of α-sheet amide II or to -N-H bend and C-N stretch of amide II [17,49]. Hence, these two bands associate with protein conformation and correlate with Raman data. The peaks at 1453 and 1401 cm^−1^ are ascribed to δ_as_(CH2, CH3) and to δ_s_(CH2, CH3) of proteins or δ(C-O) of carboxylic acids, respectively [49]. The fact that the peak at 1453 cm^−1^ gradually disappears following the order B > G > R > W could be in agreement with an effect of proteins on the acyl chains tilting in the membrane leaflet with which they are in contact, as suggested in the Raman spectra. The weak band at 1240 cm^−1^ associates with the ν_as_(P=O) stretch of diacylphosphatidylglycerol (PG) that may reflect the increased contents of this glycerolipid in G and B cells as compared to R and W cells. The bands peaking at 1152, 1080 and 1020 cm^−1^ have been shown to be representative of carbohydrates, ν(C-O-C), and thus may correspond to the polysaccharide of the external cell membrane, which would be notably altered in B cells.

## 4. Conclusions

Results shown in this study point out that *Synechococcus* sp. cells undergo changes in their glycerolipid and pigment composition in response to CCA mechanism. The variation in their PBS content and (likely) composition under the different light regimes is accompanied by variations in the thylakoid membrane glycerolipid composition. These changes involve increased content of highly saturated species of MGDG and DGDG but of the less saturated species of PG. Blue light-exposed cells accounted for the highest contents of the related glycerolipids. Differences in specific bands of Raman and ATR-IR spectra were found for the cells grown under the diverse light wavebands that can be associated with changes in protein conformation (amide I and amide II) and the biochemical composition of membranes. Differential tilt of glycerolipid acyl chains seems to be also an effect of exposure to a particular light waveband as shown by variations in the C-H bond deformation, δ(CH), band in Raman spectra and δ_as_(CH2, CH3) peak in ATR-IR spectra. Notable alteration of the polysaccharide outer membrane is likely to take place in B cells.

## Figures and Tables

**Table 1:** The ratio of the *in vivo* absorbance at 630 nm to the absorbance at 680 nm (A_630_/A_680_) for the cultures grown under the different filters. White light exposure was *ca*. 65 µmol quanta m^−2^ s^−1^. Values significantly differ between the 24 h or 48 h growth period and are indicated by the same superscript (*, **, †). W ≠ B, G or R; **, A ≠ G or R; and †, G ≠ R

Culture	A_630_/A_680_	
	24 h	48 h
Transparent filter (W)	1.02 ± 0.02^*^	1.04 ± 0.02^*^
Blue filter (B)	1.04 ± 0.03^*,**^	1.07 ± 0.04^*,**^
Green filter (G)	1.04 ± 0.02^*,†^	1.06 ± 0.02^*^
Red filter (R)	1.03 ± 0.02^*,**,†^	1.05 ± 0.05^**^

**Table 2:** The ratio of the absorbance at 475 nm to the absorbance at 665 nm for the methanolic extract of the cultures grown under the different filters. White light exposure was *ca*. 65 µmol quanta m^−2^ s^−1^. Values significantly differ between the 24 h or 48 h growth period and are indicated by the same superscript (*, **). *, W ≠ G and R; and **, B ≠ R

Culture	A_475_/A_665_	
	24 h	48 h
Transparent filter (W)	1.57 ± 0.35^*^	1.74 ± 0.42^*^
Blue filter (B)	1.45 ± 0.24	1.54 ± 0.17^**^
Green filter (G)	1.37 ± 0.33^*^	1.33 ± 0.36^*^
Red filter (R)	1.41 ± 0.31^*^	1.31 ± 0.19^*,**^

**Table 3:** Species of sulfoquinovosyldiacylglycerol (SQDG), phosphatidyldiacylglycerol (PG), monogalactosyldiacylglycerol (MGDG) and digalactosyldiacylglycerol (DGDG) detected in the UPLC-MS analysis of methanolic extracts of *Synechococcus* sp. cells. N.D. = not determined.

Compound	Chemical formulae	*m/z* for [M+NH_4_]^+^	*m/z* for [M+HCOOH-H]^−^	Fragment *m/z* from MS^E^ in negative/positive mode
SQDG (18:3/16:1)	C_43_H_74_O_12_S	832.5243	813.4850	225.005, 253.217, 277.220, 535.266
SQDG (18:3/16:0)	C_43_H_76_O_12_S	834.5406	815.4978	225.007, 255.232, 277.218 537.271, 559.256
SQDG (18:2/16:0)	C_43_H_78_O_12_S	836.5595	817.5112	225.008, 255.232, 277.218, 537.268, 561.272
SQDG (18:1/16:0)	C_43_H_80_O_12_S	838.5739	819.5295	225.005, 255.232, 281.248, 537.268, 563.291
SQDG (18:0/16:0)	C_43_H_82_O_12_S	840.5856	821.5459	225.006, 255.232, 283.268, 537.273, 565.307
SQDG (16:1/16:0)	C_41_H_76_O_12_S	810.5415	791.4961	225.009, 255.234, 253.217, 537.275, 535.356
SQDG (16:0/16:0)	C_41_H_78_O_12_S	812.5547	793.5134	225.006, 255.234, 537.275
SQDG (18:0/18:0)	C_45_H_86_O_12_S	868.6231	849.5748	225.009, 283.258, 565.312
SQDG (18:0/18:1)	C_45_H_84_O_12_S	866.6016	847.5585	225.009, 281.248, 283.258, 565.307, 563.276
SQDG (18:0/18:2)	C_45_H_82_O_12_S	864.5846	845.5427	225.001, 279.236, 283.258, 563.276
SQDG (18:0/18:3)	C_45_H_80_O_12_S	862.5696	843.5311	225.001, 277.214, 283.258, 563.276
SQDG (17:0/16:0)	C_42_H_80_O_12_S	826.5721	807.5271	225.008, 255.234, 551.284
SQDG (17:1/16:0)	C_42_H_78_O_12_S	824.5582	805.5136	225.006, 255.234, 549.274, 537.281
SQDG (17:3/16:0)	C_42_H_74_O_12_S	(820.5020)	801.4863	225.006, 255.234
SQDG (17:0/18:0)	C_44_H_84_O_12_S	854.5978	835.5544	225.006, 283.264
SQDG (17:0/18:1)	C_44_H_82_O_12_S	852.5907	833.5451	225.008, 281.248, 551.294, (563.287)
SQDG (17:0/18:3) SQDG (17:1/18:2)	C_44_H_78_O_12_S	848.5558	829.5167	(225.002), 277.217, 279.222
PG (18:3/16:1)	C_40_H_71_O_10_P	(760.5097)	741.4719	227.196, 253.217, 277.217, 505.261, 487.246
PG (18:3/16:0)	C_40_H_73_O_10_P	N.D.	743.4855	227.196, 255.233, 277.216, 505.261, 686.524
PG (18:2/16:0)	C_40_H_75_O_10_P	764.5397	745.5013	227.203, 255.233, 279.232, 507.275
PG (18:1/16:0)	C_40_H_77_O_10_P	N.D.	747.5164	227.201, 255.233, 281.248, 509.284
PG (18:1/18:1)	C_42_H_79_O_10_P	N.D.	773.5338	227.202, 281.248, 509.289
PG (17:0/18:1) PG (19:1/16:0)	C_41_H_79_O_10_P	N.D.	761.5334	227.199, 281.247, 295.259, (480.282)
MGDG (18:3/16:1)	C_43_H_74_O_10_	768.5635	795.5238	253.217, 277.217 / 773.517, 335.258, 311.259
MGDG (18:2/16:1)	C_43_H_76_O_10_	770.5809	797.5407	253.217, 279.233 / 775.533, 337.274, 311.258
MGDG (18:3/16:0)	C_43_H_76_O_10_	770.5809	797.5407	255.231, 277.217 / 775.533, 335.257, 313.274
MGDG (18:1/16:1)	C_43_H_78_O_10_	772.5952	799.5569	253.217, 281.247 / 777.549, 339.288, 311.258
MGDG (18:2/16:0)	C_43_H_78_O_10_	772.5952	799.5569	255.231, 279.233 / 777.549, 337.275, 313.274
MGDG (18:1/16:0)	C_43_H_78_O_10_	774.6095	801.5728	255.231, 281.246 / 779.566, 339.291, 313.274
MGDG (18:0/16:0)	C_43_H_80_O_10_	776.6237	803.5802	255.231, (283.255) / 781.591, 341.307, 313.273
MGDG (16:0/16:0)	C_41_H_76_O_10_	746.5768	N.D.	N.D. / 751.537, 313.274
MGDG (16:1/16:0)	C_41_H_74_O_10_	744.5636	771.5264	255.230, 253.218 / 749.517, 311.259, 313.273
MGDG (16:1/16:1)	C_41_H_72_O_10_	742.5435	(769.4965)	(253.218) / 747.501, 311.257
MGDG (18:1/18:2)	C_45_H_78_O_10_	796.5954	823.5519	279.227, 281.251 / 339.288
MGDG (18:1/18:1)	C_45_H_80_O_10_	798.6067	N.D.	281.245 / (339.292)
MGDG (18:0/18:1)	C_45_H_82_O_10_	800.6241	827.5853	281.245, 283.255 / 805.581, 339.290
MGDG (17:2/16:1)	C_42_H_74_O_10_	756.5653	N.D.	N.D. / 761.523, 311.260, 323.261
MGDG (17:3/16:0)	C_42_H_74_O_10_	756.5653	N.D.	N.D. / 761.523, 313.274, 321.243
MGDG (17:1/16:0)	C_42_H_78_O_10_	760.5928	787.5536	267.236, 255.232 / 765.550, 313.274, 325.272
MGDG (17:1/18:2)	C_44_H_78_O_10_	784.5931	811.5625	267.232, 279.233 / (789.540), 325.274, 337.275
MGDG (17:1/18:1)	C_44_H_80_O_10_	786.6086	813.5717	267.229, 281.252 / 791.563, 325.272, 339.290
DGDG (18:3/16:1)	C_49_H_84_O_15_	930.6152	957.5782	253.217, 277.217 / 935.5710, 335.258, 311.259
DGDG (18:2/16:1)	C_49_H_86_O_15_	932.6310	959.5905	255.232, 279.220 / 937.587, 337.275, 311.259
DGDG (18:3/16:0)	C_49_H_86_O_15_	932.6310	959.5905	253.217, 277.217 / 937.587, 335.258, 313.274
DGDG (18:1/16:1)	C_49_H_88_O_15_	934.6492	961.6051	253.215, 281.246 / 939.603, 339.287, 311.259
DGDG (18:2/16:0)	C_49_H_88_O_15_	934.6492	961.6051	255.232, 279.233 / 939.603, 337.275, 313.274
DGDG (18:1/16:0)	C_49_H_90_O_15_	936.6652	963.6223	255.234, 281.250 / 941.617, 339.290, 313.274
DGDG (17:3/16:1)	C_48_H_82_O_15_	916.6006	943.5618	253.214, 263.201 / 921.555, 321.242, 311.258

## Supplementary Materials

Figure S1: *in vivo* absorption spectra, Figure S2: absorption spectra of methanolic extracts, Figure S3: typical HPLC-DAD chromatogram, Figure S4: Raman and IR spectra for the two replicate cultures exposed to each waveband. Figure S5, Table S1 and Table S2 are also available in the supplementary materials which can be accessed at http://www.mdpi.com/2079-7737/5/2/22/s1.

## Abbreviations

The following abbreviations are used in this manuscript:

LHC, light harvesting complex; RC, reaction center; ROS, reactive oxygen species; CCA, complementary chromatic adaptation; PBS, phycobilisome; PBP, phycobiliprotein; PE, phycoerythrin; PC, phycocyanin; APC, allophycocyanin; chla, Chlorophyll *a*; CBRC, cyanobacteriochrome; ATR-FTIR, Attenuated Reflectance Fourier Transform Infrared Spectroscopy; FT-Raman, Fourier Transform Raman Spectroscopy; QToF, quadrupole time-of-flight (analyzer); ESI, electrospray ionization; HPLC-DAD, high performance liquid chromatography with photodiode array detection; UPLC, ultra-performance liquid chromatography; MS, mass spectrometry; EIC, extracted ion chromatogram; BPI, base peak intensity; LED, light-emitting diodes; rETRmax, maximal electron transfer rate; MGDG, monogalactosyldiacylglycerol; DGDG, digalactosyldiacylglycerol; SQDG, sulfoquinovosyldiacylglycerol; PG, diacylphosphatidylglycerol; W, white light; T, transparent filter; B, blue light; G, green light; R, red light.
