# Supplementary material for: Effect of Different Broad Waveband Lights on Membrane Lipids of a Cyanobacterium, Synechococcus sp., as Determined by UPLC-QToF-MS and Vibrational Spectroscopy
Source: Biology (Basel). 2016 May 23;5(2):22. doi: 10.3390/biology5020022 (PMC4929536; doi:10.3390/biology5020022)
Supplement: Supplementary file 1 [file biology-05-00022-s001.pdf]

Supplementary Materials: Effect of Different Broad Waveband Lights on Membrane Lipids of a Cyanobacterium, *Synechococcus* sp., as Determined by UPLC-QToF-MS and Vibrational Spectroscopy

Olimpio Montero, Marta Velasco, Aurelio Sanz-Arranz and Fernando Rull

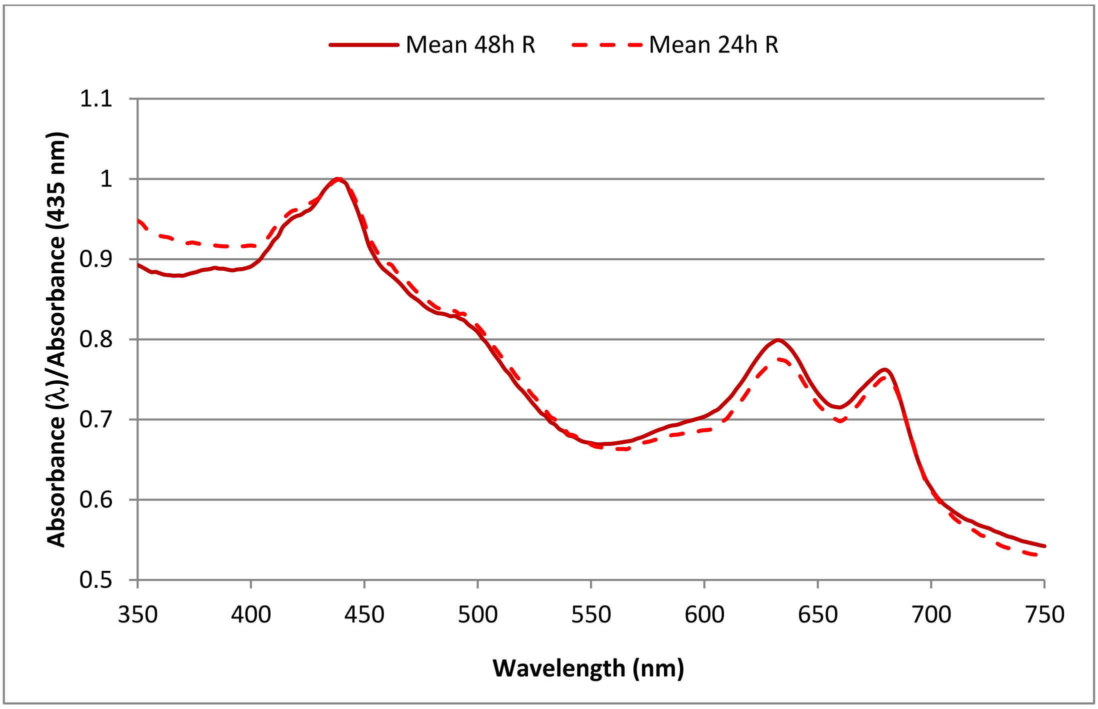

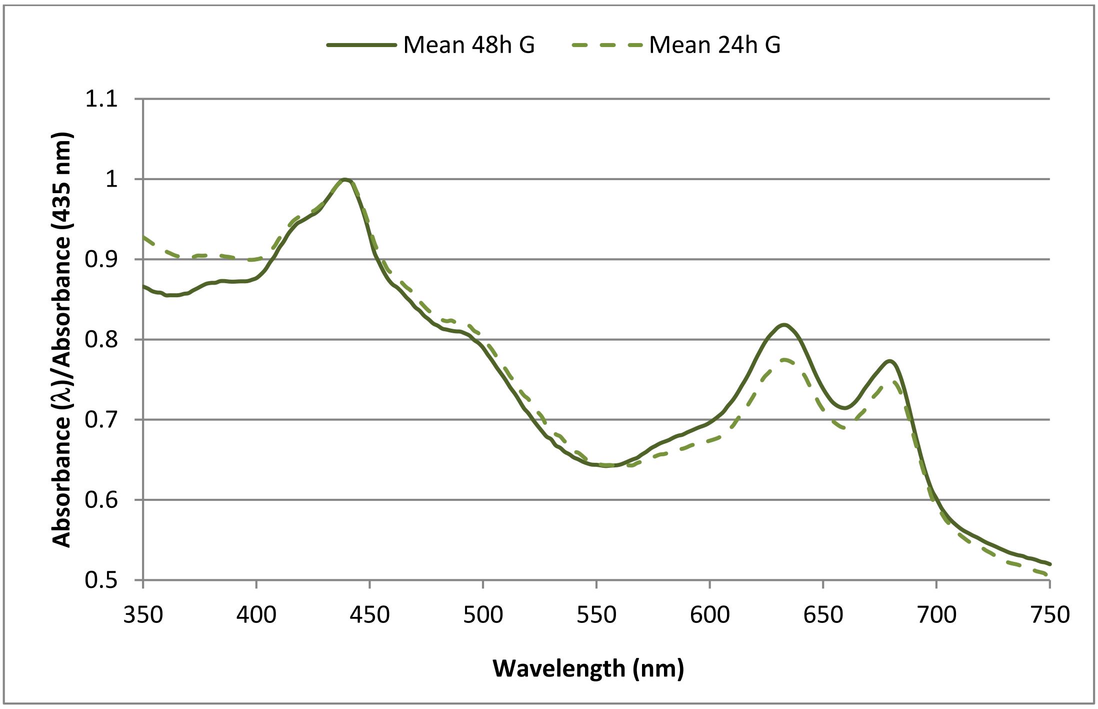

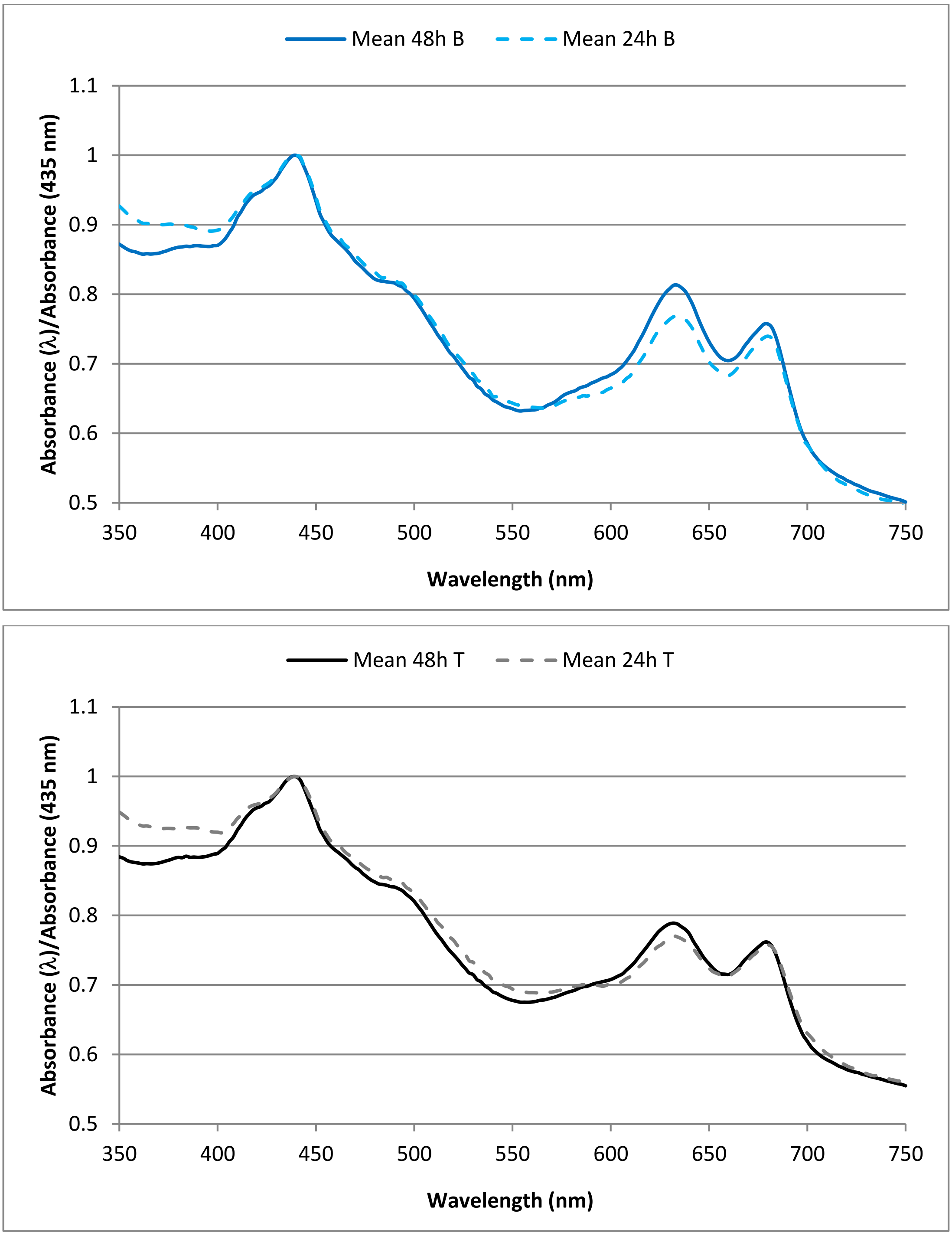

Figure S1. Comparative *in vivo* absorption spectra of *Synechococcus* sp. cells after 24h and 48h of exposure to fluorescent white light filtered by transparent filter (T, W culture), blue filter (B), green filter (G) and red filter (R). Plotted values are the mean of three independent experiments after normalization to the respective value of absorbance at 435 nm for each culture.

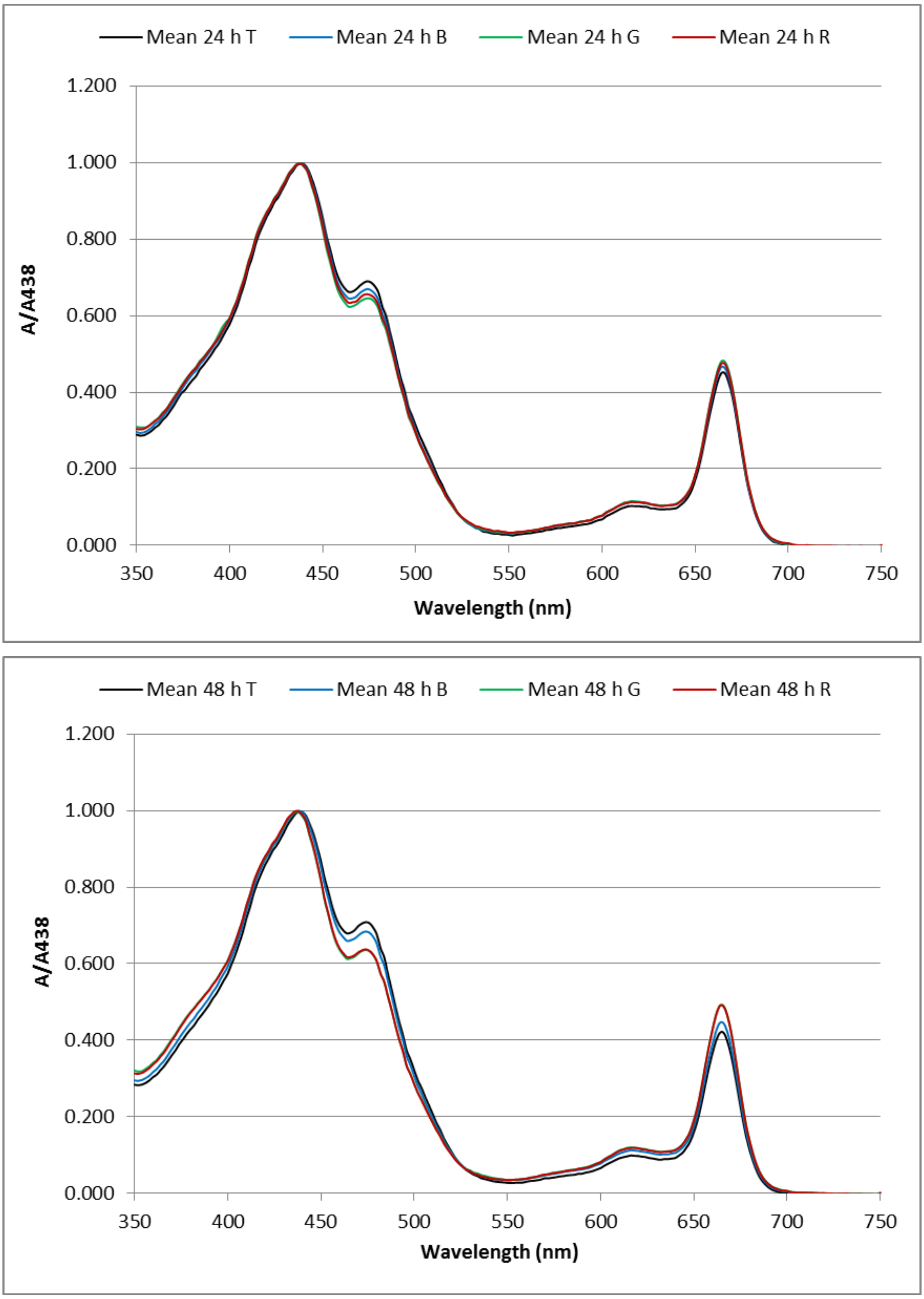

Figure S2. Comparative absorption spectra of the methanolic extracts of *Synechococcus* sp. cells after 24h and 48h of exposure to fluorescent white light filtered by transparent filter (T, white light W culture), blue filter (B), green filter (G) and red filter (R). Plotted values are the mean of three independent experiments after normalization to the respective value of absorbance at 438 nm for each culture.

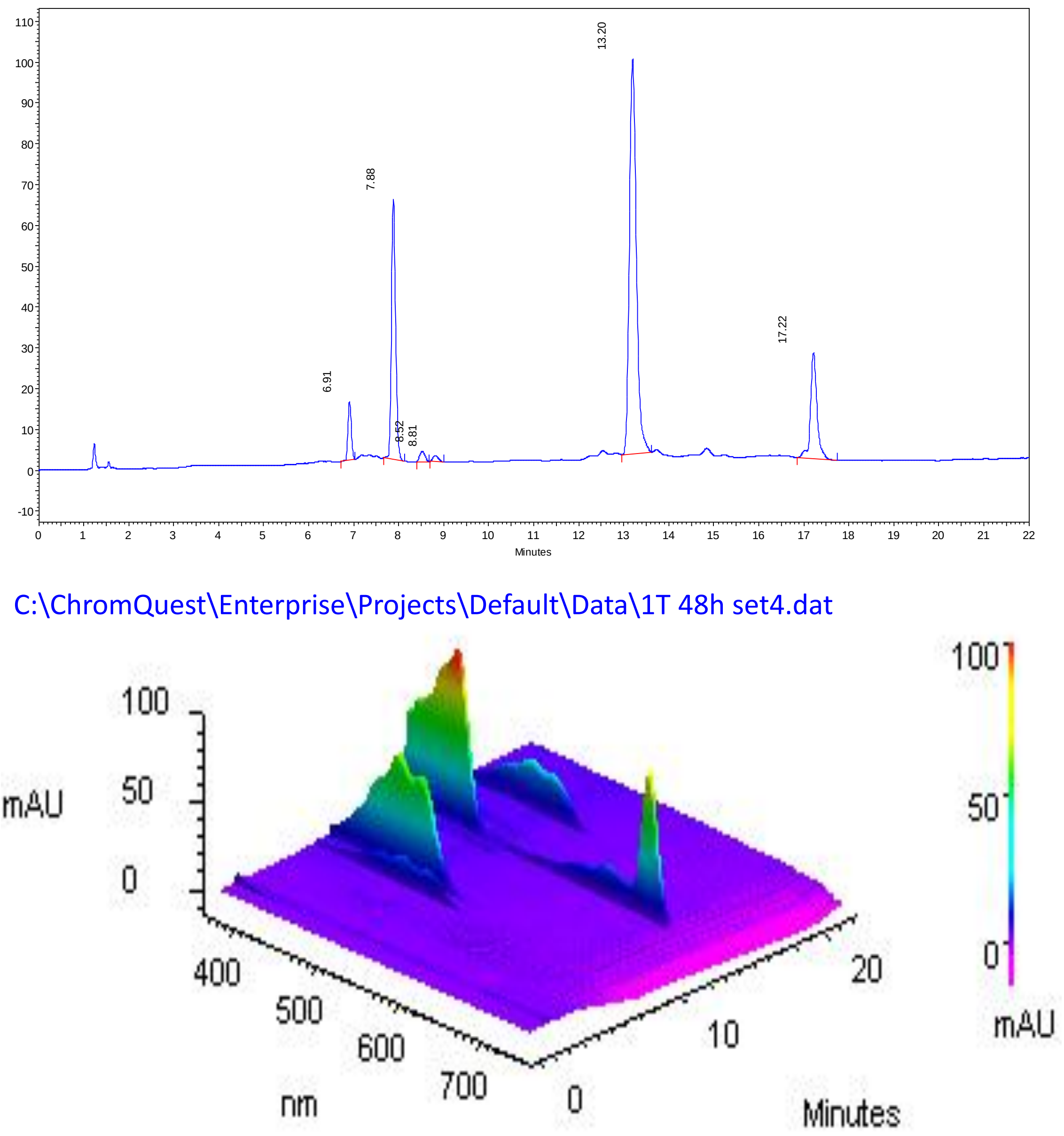

Figure S3. Typical HPLC-DAD chromatogram obtained for the methanolic extract of *Synechococcus* cells (upper panel). 3D (time-absorbance-wavelength) plot of the data acquired in a HPLC-DAD measurement (lower panel).

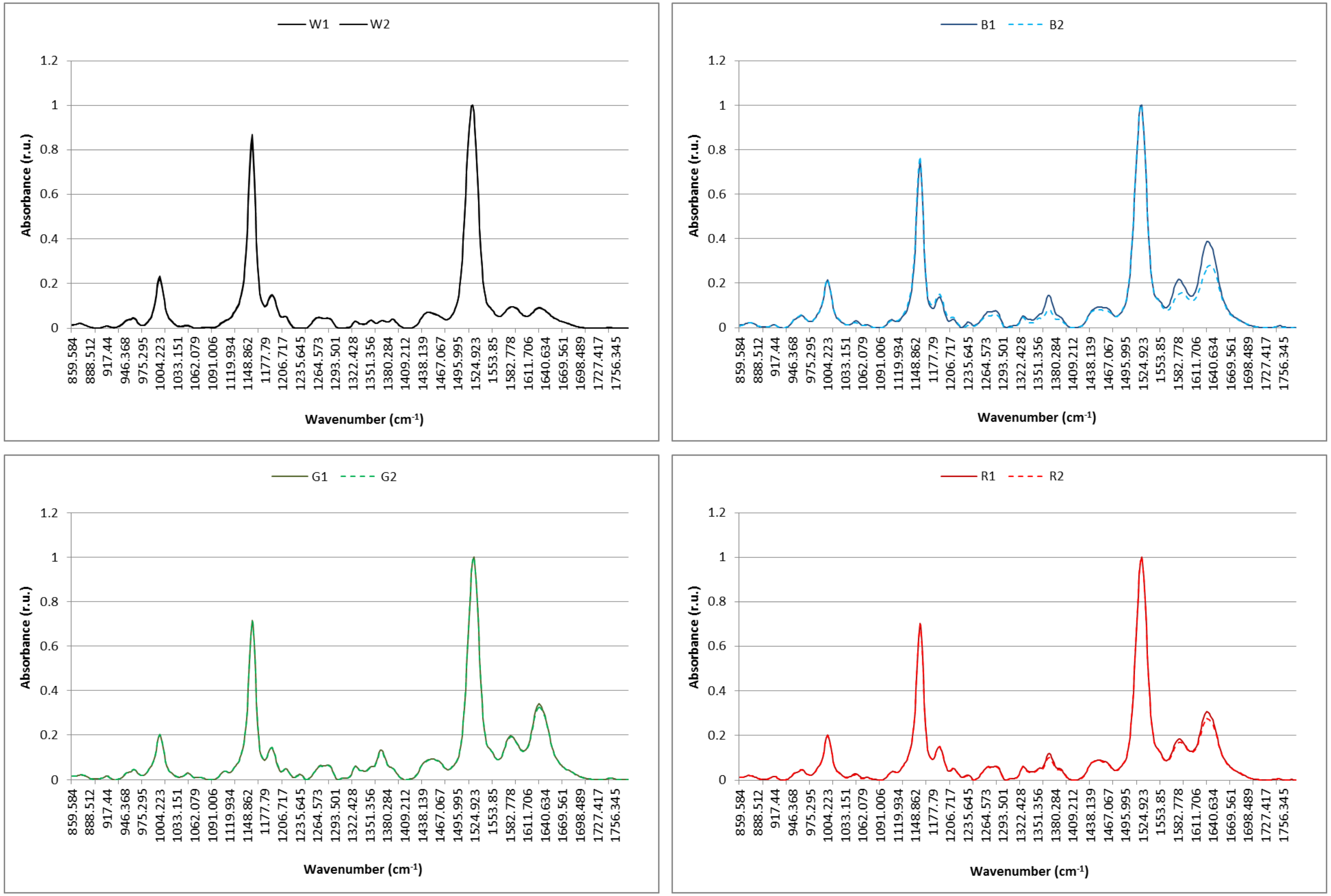

Figure S4. FT-Raman spectra of duplicates from independent experiments for each culture (W, white light; B, blue light; G, green light; R, red light).

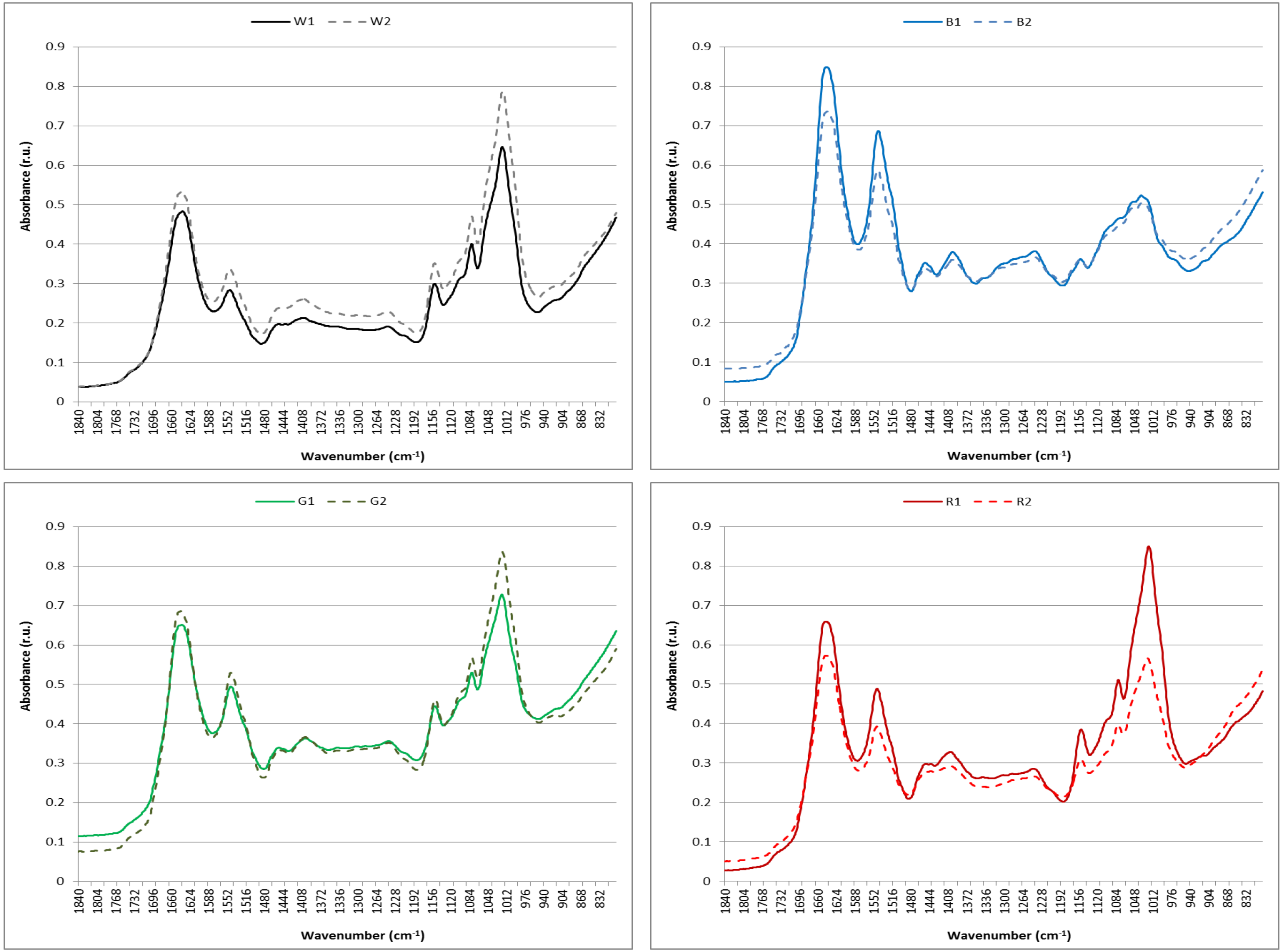

Figure S5. ATR-FT-IR spectra of duplicates from independent experiments for each culture (W, white light; B, blue light; G, green light; R, red light).

Tabla S1. Analysis of sample covariance for specific bands from ATR-FTIR spectra for the W, B, G and R cultures.

cm^{-1}	W-B	W-G	W-R	B-G	B-R	G-R
1643	−2.473E−03	−2.473E−03	7.428E−04	−1.538E−03	3.878E−03	−1.165E−03
1545	−2.333E−03	−2.333E−03	8.454E−04	−1.385E−03	4.062E−03	−1.472E−03
1453	−2.828E−04	−2.828E−04	−8.753E−05	2.878E−05	1.392E−04	4.307E−05
1401	−3.890E−04	−3.890E−04	5.862E−05	-2.036E−05	2.980E−04	−4.492E−05
1240	−3.077E−04	−3.077E−04	−7.558E−05	3.185E−05	1.516E−04	3.724E−05
1152	−8.715E−06	−8.715E−06	3.783E−04	−2.420E−06	1.219E−05	−5.290E−04
1103	−2.991E−04	−2.991E−04	4.034E−04	−1.106E−04	5.097E−04	−6.875E−04
1080	−6.208E−04	−6.208E−04	1.241E−03	−3.348E−04	1.033E−03	−2.064E−03
1020	−1.167E−03	−1.167E−03	7.696E−03	−9.054E−04	2.382E−03	−1.571E−02

Table S2. Paired t-test analysis for specific bands from ATR-FTIR spectra for the W, B, G and R cultures. (Values < 0.05 indicate statistical significant differences and are marked in red).

cm^{-1}	W-B	W-G	W-R	B-G	B-R	G-R
1643	0.024	0.024	0.017	0.105	0.076	0.183
1545	0.013	0.013	0.012	0.070	0.053	0.145
1453	0.014	0.014	0.014	0.206	0.022	0.021
1401	0.020	0.020	0.017	0.357	0.067	0.051
1240	0.008	0.008	0.009	0.075	0.008	0.007
1152	0.140	0.140	0.022	0.003	0.358	0.055
1103	0.031	0.031	0.017	0.032	0.133	0.065
1080	0.318	0.318	0.050	0.022	0.495	0.126
1020	0.045	0.045	0.273	0.018	0.141	0.337
